# Hospital Meetings, &c.

**Published:** 1897-08-07

**Authors:** 


### HOSPITAL MEETINGS, &C.

### THE ROYAL NATIONAL HOSPITAL FOR CONSUMPTION AND DISEASES OF THE CHEST, VENTNOR.

#### The Laying of the Foundation-stone for the Eleventh Block of Buildings.

H.R.H. Princess Henry of Battenberg laid the foundation-stone of the eleventh block of buildings of the Royal National Hospital for Consumption on Saturday, July 31st. The Princess, accompanied only by Miss Cochrane and Lieut. F. Ponsonby, drove to the hospital from Lansdown and was received by Sir Richard E. Webster, G.C.M.G., Q.C., M.P., chairman of the Board of Management, and other members of the Executive Committee. The president, the Right Hon. Lord Rosebery, was unfortunately not able to be present. On the Princess's arrival the band played the National Anthem, and the choir in attendance sang the same immediately after the Royal party were conducted to their seats. After this the treasurer (Mr. P. B. Burgoyne), the physician and chairman of the house committee (Dr. J. G. Sinclair Coghill, F.R.C.P.E.), the assistant physicians (Dr. Robert Robertson and Dr. John L. Whitehead, M.R.C.P.), the surgeon (Dr. James M. Williamson), the chaplain (the Rev. J. Arthur Alloway), the general superintendent (Major T. H. Khyber Payne), the matron and superintendent of nurses (Miss Busby), and the architect (Mr. Theodore R. Saunders) were presented to the Princess.

Dr. Coghill read an address of welcome, which expressed to Her Royal Highness the gratitude felt by all connected with the hospital for her kindness in consenting to lay the foundation-stone. It referred to the continual interest evinced by Her Majesty and other members of the Royal Family in the work on which they were engaged, and especially to that shown by the late Prince Henry of Battenberg. It noticed the excellence of the chosen situation, the value of the hospital as a means of saving life, and the number of inmates accommodated within its gates, and, lastly, the catholic and national lines upon which the work of the hospital was carried on.

A form of prayer, drawn up by the Bishop of Guildford, was read by the Chaplain (the Rev. J. A. Alloway), the Rector of the parish (the Rev. R. W. Odell), and the Vicar of Holy Trinity (the Rev. A. P. Clayton). During the hymn the stone was unveiled, and, all being in order, the Princess left her seat and approached it, and in her presence a sealed jar containing the coins of the realm, the Jubilee coins, a copy of the inscription on the stone, and a printed list of officers, governors, and subscribers was placed by the architect in a cavity under the foundation-stone. The Princess then spread the mortar with a silver trowel, especially designed by Messrs. Elkington, and the stone was lowered into position. The Princess gave it the traditional three taps, and, pronouncing it "well and truly laid," returned to her seat.

The Treasurer afterwards presented the silver trowel to her Royal Highness, and said that the question of ways and means was of the utmost importance, in order to make the memorial worthy of the name associated with it; and after announcing three contributions of a hundred guineas and four of £50, he concluded with: "To the end that upon the opening day this memorial may be declared free from debt I have the pleasure to contribute 2,000 guineas."

Lieut. Ponsonby read the reply to the address on behalf of the Princess, expressing her sincere thanks and appreciation of their "kindly feeling and liberality to those who had subscribed to raise so fitting a memorial to my dear husband as will be the block of buildings, the foundation-stone of which we are assembled here to-day to lay," alluding to Prince Henry's deep interest in the welfare of the hospital, and to the gratification he would have felt in its growth had he lived to witness it.

Sir Richard Webster proposed the vote of thanks to the Princess on behalf of the treasurer, staff, and patients, pointing out how valuable was the sympathy and support of the Princess both as a member of the Royal Family and as Governor of the Isle of Wight. With her approval the work was half accomplished. He concluded by calling for three cheers, which were heartily given.

The Princess then inspected Block X, and partook of refreshments before continuing her journey to Ventnor. She looked well, was dressed in widow's mourning, and undoubtedly was much pleased and gratified by the hearty welcome and affectionate testimony of sympathy offered her by her loyal subjects of the Isle of Wight.

The arrangements for the comfort of all the guests were most complete. The stand erected round the stone was guarded from the sun by awnings, and not covered by a tent. The day was perfect, the heat being tempered by a refreshing breeze. Miss Busby deserved great praise for the thoughtful care with which she provided for the Princess's comfort, for which she was entirely responsible.

The new block will cost £7,000, which is already subscribed. The foundation-stone, which is of white polished marble, bears this inscription: "To the Glory of God, and in Memory of His Royal Highness Prince Henry of Battenberg, K.G. This stone was laid by Her Royal Highness Princess Henry of Battenberg, Governor of the Isle of Wight."

